# Exploring Chia Mucilage as a Potential Additive for Salt Reduction in Traditional Balkan Minced Meat Product Ćevap

**DOI:** 10.17113/ftb.63.04.25.9071

**Published:** 2025-12-26

**Authors:** Sanja Đurđević, Igor Tomašević, Steva Lević, Nikola Stanišić, Vladimir Kurćubić, Slaviša Stajić

**Affiliations:** 1 Department of Animal Source Food Technology, Faculty of Agriculture, University of Belgrade, Nemanjina 6, 11080 Belgrade, Serbia; 2 German Institute of Food Technologies (DIL), 49610 Ouackenbruck, Germany; 3 Department of Food Technology and Biochemistry, Faculty of Agriculture, University of Belgrade, 11080 Belgrade, Serbia; 4 Institute for Animal Husbandry, Belgrade-Zemun, Autoput Beograd-Zagreb 16, 11000 Belgrade, Serbia; 5 Department of Food Technology, Faculty of Agronomy, University of Kragujevac, Cara Dušana 34, 32102 Čačak, Serbia

**Keywords:** meat product, minced meat, chia mucilage, salt reduction

## Abstract

**Research background:**

The food industry is constantly searching for solutions to reduce the sodium content in meat products as the world is facing an increased risk of diseases caused by a greater intake of sodium from salt through processed foods, including minced meat products.

**Experimental approach:**

The aim of this work is to determine potential use of chia mucilage in different mass fractions (2 and 4 %) in traditional products with reduced salt mass fraction (by 15 and 30 %) and to evaluate its impact on technological properties, colour, texture and sensory parameters of minced meat product ćevap. Given its water-binding and gelling properties, chia mucilage may exert a similar functional effect as salt in minced meat products, particularly in improving water retention and texture.

**Results and conclusions:**

The results showed that replacement of sodium chloride with chia mucilage did not have a significant effect on some technological properties, such as pH and cooking loss, but textural parameters were affected, producing softer and stickier product in general. A treatment in which sodium chloride was reduced by 15 % and 2 % chia mucilage were added was preferred in terms of appearance, juiciness and overall acceptability, while higher chia mucilage mass fractions led to lower scores in taste and saltiness perception as shown in sensory analysis.

**Novelty and scientific contribution:**

As a conclusion, it was established that chia mucilage can help reduce the salt content, but with careful reformulation so that it does not change the sensory qualities.

## INTRODUCTION

In recent years, the world has faced an increased risk of diseases caused by a high intake of sodium from salt through processed foods, including minced meat products (*1*). The recommended daily consumption for adults is less than 2000 mg of sodium, which is equivalent to less than 5 g of salt (*2*).

For this reason, the food industry is constantly searching for solutions to reduce the sodium content in meat products such as sausages, burgers and meatballs. However, given that salt plays a key role in meat products by enhancing flavour, affecting texture, as well as inhibiting microbes, reducing the salt content in meat products without compromising sensory attributes poses a significant challenge, and alternatives are difficult to find (*3*). Meat products, especially those made from minced meat (*e.g.* burgers), must be aligned with certain regulations, and for this reason the use of nitrites and phosphates is prohibited by some national regulations for some types of this product (*4*). Although nitrites contribute to microbiological safety, colour stabilization and specific taste, and phosphates improve water binding and texture, their prohibition in certain formulations emphasizes the key role of salt. This leads us to the fact that salt remains one of the main technological factors in the extraction and activation of myofibrillar proteins, improving the water binding capacity, as well as defining taste and maintaining shelf life and safety of minced meat products (*5*, *6*). Lowering salt mass fraction reduces the extracted and solubilized myofibrillar proteins, which in turn affects the technological and sensory properties of the meat system (*7*).

Recent research has shown that the incorporation of natural additives such as chia seed mucilage offers promising solutions (*3*). Chia seed mucilage is defined as a water-soluble polysaccharide obtained from the seeds of the *Salvia hispanica* L. plant through three processes: hydration, extraction, and recovery (*8*, *9*). Chia seeds contain a significant amount of dietary fibre, antioxidants including phenolic compounds, increased protein content with a balanced proportion of essential amino acids, and are rich in polyunsaturated fatty acids, especially linolenic acid (*10*). Mucilage obtained from chia seeds contains moisture, carbohydrates, protein, fat, ash and uronic acids, and has a high content of soluble dietary fibre, primarily composed of polysaccharides such as mucilage and pectin, whose presence can have health benefits by lowering cholesterol and helping intestinal functions (*11*-*13*). Due to the properties of its components, chia seed mucilage has a potential use in different food systems as a functional “clean label” (free from artificial additives, preservatives, colours or flavours) ingredient, *e.g.* texture modifier, fat replacer, stabilizer, emulsifier and others (*12*).

Until now, chia seed derivatives in meat products have been studied as a potential partial replacement for saturated fat (*10*). Due to its high content of dietary fibre, chia seed mucilage can be potentially used in meat products where fibre is used as phosphate or/and salt replacement (*14*, *15*). Such composition enables the formation of a gel, which improves water retention and modifies texture and binding capacity in food products. In addition, it shows emulsifying properties, which can improve the stability and homogeneity of minced meat formulations (*10*). Chia seed mucilage, with its hydrocolloid properties and water-holding capacity, could be a promising solution for minced meat products such as patties, meatballs and burgers. The amount of connective tissue, fat content, degree of cooking and type of heat treatment affect texture and flavour. The use of hydrocolloid components such as chia seed mucilage can be crucial, as cooking causes water loss, mass loss, and shrinkage in minced meat products (*13*, *16*). Minced meat is a widely used raw material in the production of processed meat products, including burgers, hamburgers, sausages, meatballs, and traditional Balkan dishes such as “ćevap” and “pljeskavica” (*5*, *17*). Ćevap (pronounced /t͡ɕěʋaːp/), belongs to the category of minced meat heat treated by grilling or barbecuing before consumption. This type of heat treatment affects the final product and results in changed shape, colour and taste (*5*, *6*). Given its high global consumption, there is a continuous need to enhance the quality, functionality and nutritional profile of these products. This paper investigates the use of chia seed mucilage as a potential ingredient in a traditional minced meat product from the Balkans, due to its exceptional gelling properties, potential health benefits, and possible role as salt substitute, while preserving colour, texture and sensory parameters.

## MATERIALS AND METHODS

### Chia seed mucilage preparation

Chia seed mucilage was extracted from chia seeds (*Salvia hispanica* L.) (purchased at the local market, imported from the Netherlands) using cold extraction by distilled water for 2 h as described by Hovjecki *et al.* (*18*). Extraction involved the separation of chia mucilage using an SJE 741SS juicer (SENCOR, Tokyo, Japan), followed by mixing it with 5 % *m*/*V* inulin (Cosucra, Warcoing, Belgium) as a drying aid. The mixture was dried in a laboratory oven (UF 55; Memmert, Schwabach, Germany) at 70 °C until completely dry. The dried mucilage was collected, vacuum packed and stored at 4 °C. It was ground in a Bosch KM-13 grinder (Robert Bosch GmbH, Munich, Germany) to a powdered product, which was added to meat pieces and mixed by hand with other ingredients.

### Ćevap preparation and analysis

The ćevap production process was the same as explained by Stajić *et al.* (*4*). Briefly, beef, pork (shoulder muscles) and back fat (cut into small pieces) were weighed, manually mixed with other ingredients and ground (separately) through an 8 mm plate (82H; Laska, Traun, Austria). Control treatment and four experimental treatments were prepared using 34 % beef (moisture (73.0±0.9) %, protein (21.3±0.9) % and fat (3.8±0.5) %; *N*=4 (2×2)), 34 % pork (moisture (75.1±0.8) %, protein (19.6±0.9) % and fat (4.4±0.7) %; *N*=4 (2×2)), 18 % back fat, water (11.5 %), sodium bicarbonate (0.5 %) and dextrose (0.5 %). Control sample was prepared with 1.5 % of salt, while chia seed mucilage (CM) samples were prepared as control sample but with NaCl mass fraction reduced by 15 and 30 % and with the addition of 2 and 4 % CM. In samples CM15/2, CM15/4, CM30/2 and CM30/4, the numbers denote mass fraction (in %) of NaCl reduction (first one) and mass fraction (in %) of chia seed mucilage addition (second one). Câmara *et al.* (*10*) used 2 and 4 % chia mucilage powder as phosphate replacer in emulsion-type sausage. The use of phosphates in ćevap and similar types of minced meat products is not permitted by Serbian national regulations. Therefore, we used 2 and 4 % chia seed mucilage powder as a potential partial salt replacement, considering that phosphates enhance protein solubility by disrupting the actin-myosin complex. This disruption amplifies the functional effects of salt and added water on protein extraction and solubilization, which chia seed mucilage may partially mimic through its water-binding and gelling properties. The salt amounts were based on earlier studies in which the effect of cooking loss on overall sodium content had been considered (*19*-*23*).

After refrigeration for 24 h, batches of all treatments were ground again (separately) through a 4.5-mm plate and ćevaps were formed using manual sausage feeder equipped a 20-m funnel into cylindrical shapes about 6–8 cm in length and 2 cm in diameter. After shaping, ćevaps were grilled (electric grill IEG-820; Guangzhou Ideal Catering Equipment Co., Ltd., Guangzhou, PR China) at 250 °C (75 °C in the centre), cooled at ambient temperature and kept in the refrigerator for 24 h. A total of two independent production batches were prepared, with each treatment weighing 1 kg. Between 20 and 25 individual ćevaps were obtained within each treatment (100–125 individual ćevaps for all treatments in one batch), with the average mass of (24.1±1.0) g (*N*=60; 30 per batch). The experiment was conducted in two replications on different days.

### Technological properties

The pH values were determined for 12 individual ćevaps per treatment (6 per batch) using a Testo 206 pH2 (Testo, Lenzkirch, Germany) pH meter with a penetration probe. The pH meter was calibrated before each measurement at pH=4.0 and 7.0 using standard buffer solutions. pH values were determined for both, raw and grilled products.

Six individual ćevaps per treatment (3 per batch) were used to determine water activity (*a*_w_). This was carried out using the *a*_w_ meter LabSwift-aw (Novasina, Lachen, Switzerland).

Ten individual ćevaps per treatment (5 per batch) were used to determine cooking loss (CL), which was calculated as the mass difference (in %) of raw and grilled products cooled to room temperature.

Ten individual ćevaps per treatment (5 per batch) were used to determine length reduction (LR), which was calculated as the length difference (in %) between the raw and grilled products cooled to room temperature. Digital nonius (with a 0.01 mm precision ratio) was used for measuring the length of each individual ćevap.

### Instrumental colour and texture analysis

Instrumental colour was determined on both raw (*N*=12; 6 per batch) and grilled samples (*N*=12; 6 per batch) cooled to room temperature. Colour measurements were conducted using the Computer Vision System (CVS) (*24*) with the equipment and under conditions as described by Tomasevic *et al.* (*25*). RAW photographs (files with uncompressed and unprocessed image data) of each individual ćevap surface were used to determine L*, a* and b* values of meat parts (avoiding fat parts), using a Photoshop Average Color Sampler Tool (Adobe Inc., San Jose, CA, USA). From each individual ćevap, three readings were taken on measuring area of 5×5 pixels. The average values of these measurements were calculated and used as one iteration for statistical analysis. C* (chroma) and *h* (hue angle) were calculated using the standard equations:


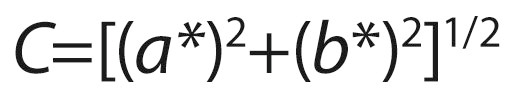
 /1/


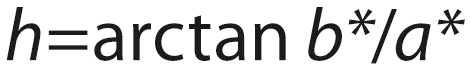
 /2/

Total colour difference (ΔE*) represents the quantification of the overall difference between two colours, *e.g.* modified treatments *vs.* control. ΔE* was calculated using the standard equation:


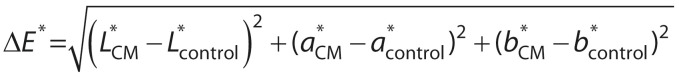
 /3/

where CM is ćevap with chia seed mucilage.

Texture profile was analysed on grilled ćevaps with the equipment (TA.XT Plus; Stable Micro System, Ltd., Godalming, UK) and under the same conditions as described by Stajić *et al.* (*4*). Six individual ćevaps per treatment (3 per batch) were held for equilibration to ambient temperature, two samples, 10 mm in height and 12 mm in radius, were taken from the centre of each individual ćevap. Hardness, adhesiveness, springiness, cohesiveness, and chewiness were evaluated and obtained using Exponent software (Stable Micro Systems).

### Sensory analysis

A preliminary sensory analysis was performed using Smart Sensory Solutions software v. 2.10.0. (*26*). Twenty untrained assessors (aged 21–60, 35 % male, 65 % female) participated in the sensory analysis and were selected based on their frequency of ćevap or pljeskavica consumption (at least once a week, or once every two weeks, based on their answers). Assessors were students (aged 21–30, 70 %) and staff members (aged 31–60, 30 %) at the Faculty of Agriculture, University of Belgrade, Serbia. Given that this type of product is usually consumed warm, the samples were heated in a microwave (GE82N-B; Samsung, Port Klang, Malaysia) for 20 s at 650 W. The temperature in the centre of the sample was about 50 °C before tasting. As the sensory analysis could not be performed on the same day, the products were prepared (by grilling), but it was necessary to heat them before serving the next day to simulate real consumption conditions. Prior to sensory evaluation, half of a ćevap from each treatment was coded with a randomly selected three-digit number, heated and served in broad daylight, at random (*N*=5). The assessors evaluated the appearance, surface colour, hardness, juiciness, odour, taste, saltiness and overall acceptability using a nine-point hedonic scale (1=extremely unacceptable, 5=neither like nor dislike, 9=extremely acceptable). Assessors used water (at room temperature) to cleanse their palates between samples. The sensory evaluations were performed in two time-separated assessments (replicates). Instructions for evaluation were briefly presented before each assessment. Due to the limited number of assessors and the preliminary nature of this analysis, the results are not shown and should be interpreted with caution.

### Statistical analysis

Statistical data processing and analysis were performed using the IBM SPSS software v. 17.0 (*27*). A one-way analysis of variance (ANOVA) and Tukey’s *post hoc* were carried out to determine significant differences among treatment groups. A level of 0.05 was used for the threshold value of significance. Results are presented as the mean value±standard deviation (S.D.).

## RESULTS AND DISCUSSION

### Effect of chia addition on techno-functional properties

Based on the statistical analysis of pH values before and after the heat treatment, as shown in Table 1, it can be inferred that the incorporation of chia seed mucilage with a concomitant decrease in sodium chloride did not result in a statistically significant (p>0.05) impact on pH values compared to the control. Antonini *et al.* (*28*) and Paula *et al.* (*29*) concluded that the addition of chia seeds does not have a statistically significant effect on the pH of meat burgers, a similar meat product to ćevap, while reduced sodium chloride content up to 33 % has no effect on pH values (*21*). These results are consistent with the research conducted by Fernández-López *et al.* (*30*), where the addition of different amounts of chia seeds to frankfurters did not have a statistically significant effect on the change in the pH value. Minor variations observed between control and treated samples may be attributed to the dissociation of ions and bioactive compounds naturally present in the chia seed mucilage, which can influence the concentration of hydrogen ions in the system (*10*). However, these variations were not statistically significant (p>0.05). Water activity (*a*_w_) values in raw samples of all treatments were higher than those observed in the control group; however, the difference was not significant (p>0.05). The observed increase in *a*_w_ may be attributed to the hydrophilic nature of chia seed mucilage, although the effect was not sufficient to produce a statistically meaningful change. Other authors reported no significant differences in *a*_w_ values, either with the same or similar types of products (*4*, *10*, *29*-*31*).

**Table 1:** Technological properties of ćevap samples

**Property**	**Control**	**CM15/2**	**CM15/4**	**CM30/2**	**CM30/4**
**pH (raw)**	(6.8±0.1)^a^	(6.88±0.08)^a^	(6.8±0.1)^a^	(6.82±0.08)^a^	(6.8±0.2)^a^
**pH (grilled and cooled)**	(7.16±0.06)^a^	(7.17±0.08)^a^	(7.1±0.1)^a^	(7.19±0.07)^a^	(7.2±0.2)^a^
***m*(CL)/%**	(19.6±1.9)^a^	(21.9±2.9)^a^	(19.8±3.0)^a^	(21.0±2.8)^a^	(22.5±2.7)^a^
**LR/%**	(16.3±2.0)^a^	(19.3±3.9)^ab^	(20.1±2.8)^b^	(23.8±3.0)^c^	(24.3±1.9)^c^
***a*_w_**	(0.96±0.00)^a^	(0.96±0.00)^ab^	(0.96±0.00)^b^	(0.96±0.00)^ab^	(0.96±0.00)^b^

Values (mean±S.D.) with different letters in superscript in the same row are significantly different (p<0.05). Numbers in chia seed mucilage (CM) samples denote mass fraction (in %) of NaCl reduction (first number) and mass fraction (in %) of chia seed mucilage addition (second number), CL=cooking loss, LR=length reduction after grilling, *a*_w_=water activity

Minced meat products, including burgers, undergo shape deformation during heat treatment. Consequently, the cooking loss and reduction in the diameter play crucial roles as technological aspects in the production of these products. Notably, in the case of a Balkan product, ćevap, the reduction in diameter is substituted by a length reduction, owning to its distinctive cylindrical shape (*4*, *5*). The reduction of sodium chloride content with the addition of chia seed mucilage had no statistically significant effect on cooking loss, but it affected the length reduction. As shown in Table 1, the result for cooking loss for CM15/4 treatment differs very little from that for the control group, a phenomenon likely influenced by the presence of chia seed mucilage and dietary fibre, which have a positive effect on water retention in the product (*3*, *28*, *32*-*34*). In contrast, in CM30/4 treatment, despite the addition of 4 % chia seed mucilage, a 30 % reduction in sodium chloride could not be fully compensated, indicating that the degree of sodium chloride reduction is the limiting factor in this case. Research on beef patties with reduced sodium, where a different amount and grain size of salt was added and its effect on cooking loss was examined, showed that more coarse salt lead to increased cooking loss, as a consequence of the lower availability of sodium ions (*35*). The reduction in length during cooking, though small, was statistically significant (p<0.05), suggesting that changes in formulation can affect product shrinkage. This may be linked to the limited ability of chia seed mucilage to compensate for the reduced amount of NaCl, which plays a crucial role in protein solubilization and structure formation.

### Instrumental evaluation of colour and texture parameters

Instrumental colour analysis of ćevap showed significant variation among treatments, associated with the mass fraction of chia seed mucilage, as shown in Table 2. In raw samples, the L* value (lightness) increased in all chia seed mucilage treatments compared to the control, with a statistically significant difference observed in CM15/4, CM30/2 and CM30/4 (p<0.05). This variation is attributed to the inherent colour of chia seed mucilage, which, in its powdered form, ranges from white to grey (*36*). Furthermore, the tabular data indicate lower a* (redness) and b* (yellowness) values in treatments with reduced salt and added chia seed mucilage. Among all treatments, CM30/4 had the lowest a* and b* values, corresponding to the highest mass fraction of chia seed mucilage and greatest salt reduction.

**Table 2:** Differences in the values of instrumental colour parameters

**Colour parameter**	**Control**	**CM15/2**	**CM15/4**	**CM30/2**	**CM30/4**
	Raw sample				
L*	(53.0±1.5)^a^	(55.341.2)^ab^	(58.4±2.0)^c^	(57.4±2.0)^bc^	(57.6±3.8)^bc^
a*	(42.4±1.7)^c^	(40.2±1.2)^bc^	(37.2±1.6)^a^	(39.1±1.4)^ab^	(36.9±2.5)^a^
b*	(14.2±1.8)^b^	(13.1±1.0)^ab^	(13.0±1.4)^ab^	(12.7±1.1)^ab^	(12.4±0.9)^a^
***C***	(44.7±2.0)^c^	(42.3±1.4)^b^	(39.4±1.8)^a^	(41.1±1.5)^ab^	(38.9±2.3)^a^
***h***	(18.4±1.8)^a^	(18.0±1.0)^a^	(19.3±1.6)^a^	(17.9±1.4)^a^	(18.6±1.8)^a^
ΔE*	/	5.6±1.8	9.2±2.8	6.6±2.8	9.6±4.6
	Grilled sample				
L*	(52.4±2.3)^b^	(47.2±2.0)^a^	(49.8±4.3)^ab^	(50.0±3.7)^ab^	(49.2±3.9)^ab^
a*	(15.9±1.43^a^	(14.8±1.5)^a^	(14.3±1.6)^a^	(14.5±0.8)^a^	(14.4±1.5)^a^
b*	(22.3±0.8)^b^	(21.0±1.5)^ab^	(19.7±2.1)^a^	(20.9±1.0)^ab^	(20.4±1.2)^a^
***C***	(27.5±1.4)^b^	(25.7±1.4)^ab^	(24.2±1.8)^a^	(25.5±1.2)^a^	(25.0±1.5)^a^
***h***	(22.3±0.8)^a^	(21.0±1.5)^a^	(19.7±2.1)^a^	(20.9±1.0)^a^	(20.4±1.23^a^
ΔE*	/	7.6±2.2	7.2±2.4	7.3±1.2	8.1±2.0

Values (mean±S.D.) with different letters in superscript in the same row are significantly different (p<0.05). Numbers in chia seed mucilage (CM) samples denote mass fraction (in %) of NaCl reduction (first number) and mass fraction (in %) of chia seed mucilage addition (second number)

After heat treatment, all samples containing chia seed mucilage showed lower L* values than control, indicating a darker appearance (Table 2). The analysis reveals a statistically significant difference (p<0.05) between CM15/2 and the other treatments, among which no statistically significant differences were detected. The results differ from earlier research that looked at the use of chia seed mucilage as a supplement or substitute for various meat components. Specifically, research showed that the inclusion of chia seed mucilage as a fat replacer in beef patties increased lightness (L*) and yellowness (b*) (*13*, *33*). However, a decrease in redness (a*) was observed with the addition of chia seed mucilage (*13*). Research with Bologna sausages and a model system emulsion led to a reduction in L* and a* colour values (*10*). Similarly, Pintado *et al.* (*3*) and Fernández-López *et al.* (*30*) obtained comparable results, noting that the incorporation of chia flour resulted in decreased L* and a* values. The observed colour changes in other types of products are likely associated with variations in moisture and fat content, which influence light reflectance and, consequently, the brightness of the product. However, this phenomenon does not apply to the current research, as there are no variations in the composition of the treatments. Furthermore, the variation in cooking loss is minimal and does not affect the chemical composition. The observed decrease in L* and a* values may be attributed to the unique chemical composition of chia seeds, which enhances their water-binding capacity (*30*). Although a* values (redness) do not show a statistically significant variation, it is noteworthy that samples with higher chia seed mucilage mass fraction, specifically CM15/4 and CM30/4, showed numerically lower a* values, indicating reduced redness. Moreover, the b* values (yellowness) demonstrate statistically significant differences between samples with the highest chia seed mucilage mass fraction and control, with the former displaying lower b* values and thus appearing less yellow (Table 2).

The ΔE* values, which indicate total colour difference of modified treatments compared to control, show that raw samples with 4 % chia seed mucilage are more different than samples with 2 % chia seed mucilage powder. On the other hand, this was not the case after grilling, as all ΔE* values were within a narrow range, between 7 and 8. Salt reduction did not affect ΔE* values in either raw or grilled ćevap. The observed ΔE* values were not higher than 10, indicating that colour differences in the modified treatments (compared to control) will probably be noticeable, considering that Djekic *et al.* (*37*) highlighted perceptible differences within the 2–10 range.

Textural properties are crucial in determining the quality and sensory properties of food products. Table 3 shows the results of texture profile analysis for ćevap samples formulated with a reduced sodium content (by 15 and 30 %) and varying mass fractions (2 and 4 %) of added chia seed mucilage, highlighting the impact on parameters such as hardness, cohesiveness, springiness, chewiness and resilience. The presented results indicate that the reduction of the salt content together with the addition of chia seed mucilage had an effect on the reduction of hardness. Namely, all modified treatments had a lower hardness value and did not differ statistically from control. Research on beef patties showed similar results, where the addition of chia seeds resulted in reduced hardness and chewiness of these products (*13*, *32*). Fernández-López *et al.* (*30*) obtained the same results with the experiment on frankfurters, where the addition of chia seed mucilage in the form of flour resulted in reduced hardness of the product. Similar results were also reported by Arifin *et al.* (*38*) and Barros *et al.* (*39*). The addition of other plant-based ingredients, such as pitahaya peel flour, also negatively affected textural parameters by reducing the hardness, chewiness and gumminess in products similar to ćevap (*40*). The analysis shows that only CM15/2 had no significant differences compared to control. Therefore, it can be concluded that the addition of chia seed mucilage powder even in higher amounts could not compensate for the reduction of the salt content. The decrease in hardness may be due to the gel matrix of the chia seed mucilage, namely the hydrophilic proteins and soluble fibre in the mucilage that keep water bound and result in softer structure (*13*, *32*). Sample CM30/4 shows the highest value for adhesiveness, indicating it is the stickiest of the treatments. Conversely, control sample shows the lowest adhesiveness, suggesting that salt reduction increases the stickiness of the samples. Reducing the salt content may result in decreased springiness values, as demonstrated in this research. Specifically, greater mass fractions of salt reduction were associated with lower springiness values, and the addition of chia seed mucilage was insufficient to counteract this effect. Câmara *et al.* (*31*) showed that 5 % of chia seed mucilage decreases springiness and cohesiveness of the meat model system, potentially because of dietary fibre inhibiting the aggregation of myosin globular heads, which is the initial stage in the protein gelation that occurs at high temperatures. CM15/2 shows the highest springiness among all treatments, although the difference is minor. CM15/4 and CM30/4 had the lowest cohesiveness (*31*, *33*), indicating poorer internal cohesion after the addition of 4 % of chia seed mucilage. Also, the same amount of added chia seed mucilage in CM15/4 and CM30/4 showed the lowest resilience, reflecting the reduced recovery after deformation. Chewiness was also significantly affected, with all chia seed mucilage enriched and salt-reduced treatments, showing lower values and reflecting a softer and less cohesive texture. The results indicate that all four treatments consistently show lower values across most textural parameters, suggesting the treated samples are softer, stickier and less cohesive.

**Table 3:** Results of the texture profile analysis

**Property**	**Control**	**CM15/2**	**CM15/4**	**CM30/2**	**CM30/4**
**Hardness/N**	(1887±199)^c^	(1745±152)^bc^	(1439±84)^a^	(1573±111)^ab^	(1566±220)^ab^
**Adhesiveness/(N·s)**	(-13.5±7.9)^a^	(-8.8±8.4)^ab^	(-9.4±7.6)^ab^	(-7.8±6.9)^ab^	(-4.0±6.4)^b^
**Springiness**	(0.87±0.02)^bc^	(0.88±0.03)^c^	(0.83±0.04)^a^	(0.84±0.02)^ab^	(0.84±0.04)^ab^
**Cohesiveness**	(0.67±0.03)^b^	(0.64±0.06)^b^	(0.55±0.05)^a^	(0.57±0.05)^a^	(0.55±0.03)^a^
**Chewiness/N**	(1104±120)^b^	(981±142)^b^	(664±94)^a^	(749±64)^a^	(716±98)^a^
**Resilience**	(0.31±0.02)^b^	(0.28±0.03)^b^	(0.23±0.03)^a^	(0.24±0.03)^a^	(0.23±0.02)^a^

Values (mean±S.D.) in the same row with different superscripts are significantly different (p<0.05). Numbers in chia seed mucilage (CM) samples denote mass fraction (in %) of NaCl reduction (first number) and mass fraction (in %) of chia seed mucilage addition (second number)

### Descriptive sensory evaluation

A preliminary sensory evaluation was conducted to provide indicative insight into the acceptability of products with different mass fractions of sodium chloride reduction and the addition of chia seed mucilage across attributes such as appearance, colour, hardness, juiciness, odour, taste, saltiness, and overall acceptability. Due to the small number of assessors, the results are not shown and should be interpreted only as an initial orientation, not as conclusive findings (data not shown).

The appearance scores showed that the formulation with a 15 % reduction in NaCl and the addition of 2 % chia seed mucilage achieved the most desirable rating. This suggests that this combination may enhance the visual appeal of the meat product, potentially due to the ability of mucilage to improve moisture retention and binding properties (*12*). In contrast, both formulations with a 4 % chia seed mucilage addition received lower scores. Although there is a distinction between CM15/2 and CM15/4, we cannot conclude that salt mass fraction reduction combined with the addition of chia seed mucilage significantly alters the exterior appearance. Colour ratings were consistent, and remained high across all samples, with no significant differences observed (p>0.05). This indicates that the incorporation of chia seed mucilage, regardless of NaCl mass fraction reduction, does not adversely affect the colour, which is essential for the perception of freshness and quality in meat products. The same data for colour were obtained using a gel emulsion prepared with chia seeds and olive oil in beef patties (*33*). Hardness scores were similarly consistent, with no significant differences among the samples. This suggests that the structural integrity of the meat products is maintained even with the addition of chia seed mucilage, which is crucial since texture is a key determinant of sensory panel acceptance and preference. The sensory panel rated the treatment with a lower amount of NaCl reduction and lower addition of chia seed mucilage, particularly in CM15/2, highest in terms of juiciness, although hardness and cohesiveness were reduced. However, the flavour profile appears to be compromised in CM30 formulations, where a decline in juiciness scores was observed. The results indicate that either the sensory panel did not notice the differences determined by instrumental texture measurement or if they did, they did not consider them negative. Yüncü *et al.* (*32*) and Arifin *et al.* (*38*) reported an increase in juiciness scores, which does not correlate with other results (*29*). This indicates that while chia seed mucilage can enhance perceived juiciness, higher salt reduction may lead to a less palatable experience when combined with excessive mucilage. Odour scores remained high and consistent, suggesting that the addition of chia seed mucilage does not negatively affect the aromatic profile of the meat products, which is in agreement with Liu *et al.* (*33*). This is an important finding, as the olfactory properties of meat significantly impact overall consumer acceptability. Taste perception varied notably, with CM15/2 and CM30/2 maintaining higher scores. In contrast, both samples with 4 % chia seed mucilage addition had lower taste ratings, particularly CM30/4. This suggests that while reducing NaCl can be beneficial for health considerations, excessive reduction paired with higher chia seed mucilage may lead to an undesirable taste profile, potentially due to the distinct flavour or texture of mucilage altering the inherent taste of meat (*30*, *32*, *33*). Saltiness perception was highest in the control and CM15/4, and decreased in the CM30 formulations, particularly in CM30/4. This finding highlights the importance of salt in flavour enhancement and may suggest that while chia seed mucilage can help reduce sodium content, it does not replicate the taste impact of salt effectively at higher mucilage mass fractions. Overall acceptability mirrored the trends observed in other sensory attributes, with CM15/2 achieving the highest score and CM30/4 the lowest. This illustrates that while chia seed mucilage offers an innovative means of reducing sodium content in meat products, the optimal formulation must balance health benefits with sensory quality.

## CONCLUSIONS

Adding 2 and 4 % chia seed mucilage powder, along with reducing salt content by 15 and 30 %, altered the technological properties of the Balkan minced meat product ćevap. Treatments with greater salt reduction and higher amounts of chia seed mucilage powder were more prone to deformation during grilling. Raw modified treatments were lighter and less red and yellow than the control. However, this difference was not observed after grilling. Regarding instrumental texture, adding chia seed mucilage powder did not compensate for salt reduction, as modified treatments had lower hardness and chewiness and were less elastic and cohesive. Although a preliminary sensory analysis was conducted, the small number of assessors limits the strength of the conclusions. Nevertheless, early indications suggest that the altered technological properties were either not always noticeable or were not perceived negatively. Notably, the optimal balance appears to be achieved with a 15 % reduction in salt combined with a 2 % addition of chia seed mucilage, enhancing overall acceptability without compromising flavour or texture. This study underscores the potential of natural additives like chia seed mucilage in reformulating traditional minced meat products and promoting healthier consumption while respecting culinary traditions. Future research should further investigate the long-term effects of such formulations on product stability and consumer preferences, paving the way for broader applications in the meat industry.
